# Forging the Frontiers of Image-Guided Neurosurgery—The Emerging Uses of Theranostics in Neurosurgical Oncology

**DOI:** 10.3389/fbioe.2022.857093

**Published:** 2022-07-12

**Authors:** Fred C. Lam, Uyanga Tsedev, Ekkehard M. Kasper, Angela M. Belcher

**Affiliations:** ^1^ The David H. Koch Institute for Integrative Cancer Research, Massachusetts Institute of Technology, Cambridge, MA, United States; ^2^ Division of Neurosurgery, Saint Elizabeth’s Medical Center, Brighton, MA, United States; ^3^ Department of Biological Engineering, Massachusetts Institute of Technology, Cambridge, MA, United States

**Keywords:** theranostic, neurosurgery, fluorescence-guide surgery, nanotechnology, NIR imaging *in vivo*

## Introduction

### A Brief History of Neurosurgical Oncology

Modern neurosurgical treatment of brain tumors involves maximal safe resection of tumorous tissue whilst preserving adjacent normal neurovascular anatomy in order to minimize the risk of neurological injury to the patient (Sunaert, 2006). A surgical series of 8 patients performed by renowned Johns Hopkins neurosurgeon Dr. Walter Edward Dandy, published in 1933, involved surgical removal of the right cerebral hemisphere with preservation of the right basal ganglia, in right-handed patients who presented with left-sided paralysis due to their right hemispheric tumors (Dandy, 1933). This pioneering report demonstrated the invasiveness of malignant brain tumors despite heroic surgical efforts, as patients inevitably succumbed to residual disease in the preserved basal ganglia, with invasion into the opposite hemisphere, emphasizing both the need for better visualization of tumor cells within the brain during surgery and the necessity for adjuvant chemotherapy and radiation therapy to treat residual tumor burden. Further pioneering work throughout the past century by other neurosurgical greats including: Drs. Harvey Cushing, William Halsted, Fedor Krause, Victor Horsley, Wilder Penfield, Gazi Yaşargil, Rolando Del Maestro, and others, paved the way for the advent of cortical brain mapping techniques, use of the operating microscope, and stereotactic surgery—all of which are now common tools in the neurosurgeon’s armamentarium to enable safe surgical resection of brain tumors (exquisitely reviewed in “A History of Neuro-Oncology,” by Dr. Rolando Del Maestro) (Del Maestro, 2006).

Further pioneering work published in 1916 by Dr. Dandy and his then senior neurosurgery resident, Dr. George J. Heuer, reported the localization of brain tumors in one hundred consecutive patients using x-ray roentgenography (Heuer and Dandy, 1916). This was followed by a series of studies by Dr. Dandy in which he injected air, and later, contrast agents, into the ventricular and lumbar subarachnoid spaces to diagnosis and localize brain and spinal tumors using x-rays (Dandy, 1918; Dandy, 1919). The herculean efforts of renowned neurologist Dr. Egas Antonio Ceatano de Moniz and neurosurgeon Dr. Pedro Almeida Lima in Lisbon, Portugal, in developing the technique of cerebral angiography for the localization and visualization of the blood supply of brain tumors, led to Dr. Lima’s documentation of over 2000 angiograms in 1949, paving the way for the indispensable use of cerebral angiography in modern day neurosurgery for the diagnosis and treatment of brain tumors and the coiling of cerebral aneurysms (Moniz, 1927; Del Maestro, 2006).

### Intraoperative Image-Guided Technology in Neurosurgical Oncology

The use of intravenous fluorescein tagged with radioactive ^131^iodine to differentiate tumor tissue from normal brain under ultraviolet (UV) light by Dr. George Eugene Moore in 1947 at the University of Minnesota was one of the first reports of exploiting the inherent leakiness of the blood-brain barrier (BBB) around brain tumors to allow for the extravasation of a radioactive fluorophore to enhance visualization of brain tumors. This landmark discovery paved the way for the introduction of radioactive nucleotides such as ^18^fluorodeoxyglucose for use with computed tomography (CT) and positron emission tomography (PET) to localize metabolically active tumor cells in the brain. However, it was the advent of nuclear magnetic resonance (NMR) by Professors Felix Block from Stanford University and Edward Purcell from Harvard, for which they were awarded the Nobel Prize in Physics in 1952, that led to the introduction of magnetic resonance imaging (MRI) scanners, which has since revolutionized our ability to visualize with stunning resolution the neurovascular anatomy of the brain and spinal cord. Since then, adaptations such as functional MRI (fMRI) (Ogawa et al., 1990; Kwong et al., 1992) and diffusion tensor imaging (DTI) (O'Donnell and Westin, 2011; Alexander et al., 2007) have allowed for the neurosurgeon to superimpose multiple layers of imaging information on top of contrast agent enhanced MRI sequences to allow for rendering of the spatial characteristics of brain tumors in eloquent regions of the brain (using fMRI) with respect to neighboring neuronal fiber tracts (using DTI) to achieve maximal safe resection of brain tumors (Algethami et al., 2021).

Incorporating different image-guided technologies into the operative neurosurgical workflow varies depending on the modality that is used. Many neurosurgical centers now have access to portable neuronavigation systems such as the Medtronic StealthStation™ (Minneapolis, MN, United States) that occupy minimal footprint in the operating room and can overlay high resolution CT or MRI imaging with fMRI or DTI images to allow for fixed 3D mapping of intracranial lesions for presurgical planning Algethami et al. (2021). The disadvantage of these fixed imaging systems is that they do not allow for real-time evaluation of intra-operative resection margins, unlike intraoperative MRI (iMRI), which currently provides the highest quality resolution of EoR and assessment of changes in the brain throughout the course of a tumor resection (Rogers et al., 2021). However, given the large footprint required to accommodate an MRI scanner, the need for specialized operating rooms with MRI compatible (non-ferromagnetic) instruments that are required to allow for positioning of the patient in and out of the scanner whilst providing adequate surgical access throughout the course of the operation, and the high costs associated with purchasing and installation of the MRI scanner and the MRI compatible instruments, the use of iMRI is currently limited to specialized neurosurgical centers that can afford this technology (Mislow et al., 2009; Chicoine et al., 2011).

Despite the improvements in preoperative imaging planning workflow, the surgeon is often times limited in their ability to differentiate tumor tissue from adjacent normal neuroanatomy under direct white light microscopy. This is particularly challenging during resection of infiltrative tumors such as high-grade gliomas, the most common and aggressive primary brain tumor in adults. To overcome this limitation, fluorescence-guided surgery (FGS) using tumor-targeting fluorophores have steadily been introduced into the neurosurgeon’s armamentarium (Vahrmeijer et al., 2013). The FDA-approved protoporphryin IX prometabolite 5-aminolevulenic acid (5-ALA) has yielded promising results in increasing the EoR for high-grade gliomas (Stummer et al., 2006; Della Puppa et al., 2014a; Belloch et al., 2014), but has shown no clear benefit in lower-grade disease where tumor cells are less metabolically active, thereby decreasing the levels of intraoperative fluorescence to allow for maximal safe EoR (Mirza et al., 2022). Furthermore, the need to orally administer 5-ALA to the patient 2.5–3 h prior to induction of anesthesia followed by strict avoidance of direct exposure to sunlight or bright room lights for 24 h after 5-ALA administration due to increased skin photosensitivity (Tonn and Stummer, 2008) and the relative high cost of 5-ALA, have limited its widespread use. Alternatively, the FDA-approved fluorescent dye sodium fluorescein, which has been widely used and tested for safety in the field of ophthalmology, is inexpensive, can be administered intravenously at the time of surgery, and relies on the leakiness of the surrounding BBB to accumulate at the site of high grade brain tumors (Acerbi et al., 2014; Cavallo et al., 2018). Unlike 5-ALA, fluorescein does not depend on the metabolic activity of tumor cells and could lead to false-positive identification of non-tumorous cells surrounding the leaky BBB, however several studies comparing fluorescein-guided to white light resections have demonstrated a high level of accuracy in identification of tumor tissues (Kuroiwa et al., 1998; Kuroiwa et al., 1999; Acerbi et al., 2013; Acerbi et al., 2014). The much lower cost of fluorescein and its safety profile supported by the ophthalmologic literature have increased its prevalence for use in neurosurgical procedures (Kwiterovich et al., 1991; Kwan et al., 2006). Disadvantages of 5-ALA and sodium fluorescein are that while both fluorophores can clearly demarcate tumor boundaries, they lack depth of tissue penetration with laser excitation in their respective wavelengths and their signal can be masked by endogenous cellular autofluorescence (Acerbi et al., 2014; Mirza et al., 2022). More recently, the FDA-approved near-infrared fluorophore indocyanine green (ICG), traditionally used in angiography procedures, has gained popularity in FGS. Like fluorescein, ICG also accumulates at the tumor site through leaky BBB vasculature but has also been shown to achieve endocytosis inside tumor cells (Onda et al., 2016), whereas fluorescein does not, allowing for higher sensitivity of detection of tumor cells during surgery which correlates well with gadolinium contrast-enhancing tumor signal on preoperative MRI (Lee et al., 2016).

### Evidence for Using Intraoperative Imaging Technology to Maximize Extent of Resection to Improve Survival Outcomes in Neurosurgical Oncology

Successful outcomes in neurosurgical oncology is increasingly being defined by the EoR to achieve a gross total resection (GTR) or maximal safe cytoreduction (Rogers et al., 2021). Several studies have reported the EoR as the single most significant variable for impacting survival in patients with newly diagnosed or recurrent high grade gliomas (HGG) (Lacroix et al., 2001; Sanai et al., 2011; Oppenlander et al., 2014). Similarly, a GTR or maximal safe EoR were associated with significantly longer progression free survival (PFS) and overall survival (OS) in patients with low grade gliomas compared to performing a biopsy alone (Roelz et al., 2016; Scherer et al., 2020).

Studies comparing across image-guided surgical modalities demonstrated that iMRI was superior over both 5-ALA FGS and conventional neuronavigation approaches in obtaining GTRs and led to prolonged survival (Knauth et al., 1999; Bohinski et al., 2001; Senft et al., 2011; Golub et al., 2020). A recent Cochrane Database network meta-analysis comparing image-guided technologies to maximize EoR for resection of gliomas yielded a paucity of high level evidence supporting the use of iMRI or 5-ALA in achieving maximal EoR with heavily biased results and inconclusive evidence regarding improvements in PFS and OS (Fountain et al., 2021). Furthermore, a brief cost benefit analysis suggested that image-guided surgery, iMRI, and 5-ALA FGS in particular, are associated with increased costs compared to conventional surgical resections, and therefore further research and randomized controlled trials will be needed in order to determine whether these modalities should be offered as standard of care for resection of brain tumors (Fountain et al., 2021). A more recent frequentist network meta-analysis was published identifying 23 studies with 2,643 patients comparing 5-ALA, sodium fluorescein, and iMRI, to no image guidance for the resection of HGGs (Naik et al., 2022). This study found that image guidance using iMRI, fluorescein, and 5-ALA led to greater rates of GTR, improved PFS, and OS compared to no image guidance. However, both meta-analyses confirmed that future studies are needed to assess superiority between modalities as well as other metrics including duration of surgery while using image guided techniques and cost.

One caveat that must be taken into consideration when interpreting the results of the aforementioned network meta-analysis is that the majority of studies using 5-ALA to maximize EoR were in patients with HGGs that were located in the supratentorial space (i.e. Frontal, parietal, occipital, and temporal lobes of the brain) (Stummer et al., 2006). These are regions of the brain that are relatively easy to access surgically, and patients can generally tolerate aggressive tumor tissue debulking without significant neurological sequelae. Thus, conventional neurosurgical approaches are more likely to yield EoR margins and outcomes that are comparable to image-guided approaches. However, lesions that are located in more sensitive regions of the brain (i.e. the brainstem, posterior fossa) may benefit from image-guided surgery in order to better appreciate tumor margins around brainstem nuclei and nerve fiber tracts that control essential neurological and life-sustaining functions while achieving maximal safe EoR (Della Puppa et al., 2014b; Della Puppa et al., 2015; Algethami et al., 2021). In fact, the importance of intraoperative visualization for maximal safe brain tumor resection has been recognized by the global neurosurgical oncology community, as evidenced by the inaugural 2021 conference on intraoperative visualization and the connectome, organized by the prestigious Society for Neuro-Oncology (https://www.soc-neuro-onc.org/WEB/WEB/Event_Content/Intraoperative_Visualization_and_the_Connectome.aspx).

### The Emerging Uses of Nanoscale Materials to Augment Fluorescence-Guided Neurosurgery

Forging the frontiers of image-guided neurosurgery is the emerging use of nanoscale materials for the detection and treatment of tumor cells in neurosurgical procedures. Nanoscale materials are self-assembling polymeric systems measuring less than 1,000 nm in their longest axis. These systems can be organic or inorganic in nature, and can be functionalized with targeting moieties to facilitate delivery across the BBB into the CNS with the ability to then deliver payload to specific cells of interest in the brain. Nanoparticles (NP) that have been developed for potential CNS applications typically have a maximal diameter of ∼100 nm to facilitate trafficking across the tight junctions of the BBB. We previously developed a liposomal NP of ∼100 nm in diameter that was functionalized with a fluorophore and transferrin to enable transferrin receptor-mediated transcytosis across the BBB to deliver dual combination therapies to glioma brain tumors in mice (Lam et al., 2018). Other researchers have developed mesoporous silica NPs, magnetic iron oxide NPs, gold NPs, copolymers, and carbon nanotubes, for delivery of cargo into the brain (a summary of these nanoplatforms with accompanying references are provided in Table 1). Functionalization of NPs with surface moieties such as transferrin, folate, cyclic RGD peptide, or angiopep-2, that enable trafficking across the BBB and targeting to tumor cells *in vivo* can improve specificity of delivery, although the effectiveness of receptor targeting of NPs in solid tumors has been recently questioned (Obaid, 2021 #233). We have provided a tabular summary of these NP formulations, specific targeting ligands and sequences, along with potential applications for use in neurosurgical oncology for ease of reference for our readers (Table 1).

**TABLE 1:** Summary of different physicochemical and functional properties of nanoparticles with their potential clinical applications in neurosurgical oncology.

Types of nanoparticles	Liposomes/polymersomes Jiang et al. (2012), Zong et al. (2014), Shi et al. (2015)
	Iron oxide NP Alphandéry et al. (2015)
	Silica NP Wang et al. (2015)
	Gold NP Fan et al. (2014)
	Quantum dots Onoshima et al. (2015)
	Carbon nanotubes Wang et al. (2012), Ceppi et al. (2019)
	Bacteriophage Ghosh et al. (2012), Yi et al. (2012), Staquicini et al. (2020), Wang et al. (2021)
	Copolymers Ke et al. (2017)
BBB trafficking receptors, peptide sequences, and ligands	Mannose Du et al. (2014)
	Glucose Du et al. (2014)
	Lactate Pérez-Escuredo et al. (2016)
	Neutral amino acids Nawashiro et al. (2005), Kobayashi et al. (2008), Abbott et al. (2010), Geier et al. (2013)
	Cationic amino acids Barar et al. (2016)
	Anionic amino acids Smith (2000), Hawkins et al. (2006)
	Oligopeptides and polypeptides Butte et al. (2014), Dardevet et al. (2015)
	Transferrin Wang et al. (2009), Zhou et al. (2021)
	Folate Wang et al. (2009), Zhou et al. (2021)
	LRP1 Jiang et al. (2018)
Tumor targeting peptide sequences, ligands, and receptors	RGD Qin et al. (2011), Sharma et al. (2013), Zong et al. (2014), Liu et al. (2015), Shi et al. (2015), Wang et al. (2016), Joshi et al. (2017), Ke et al. (2017), Yao et al. (2017)
	NGR Sharma et al. (2017)
	CGKRK Sharma et al. (2017)
	Angiopep-2 Gao et al. (2014)
	Chlorotoxin Veiseh et al. (2009), Wang et al. (2020)
	HA Jiang et al. (2012)
	CD133 Jing et al. (2016)
	Folate Cho et al. (2019)
	Transferrin Dixit et al. (2015)
	LRP1 Jiang et al. (2018)
Potential clinical applications	Drug delivery to brain tumors d'Angelo et al. (2019), Dixit et al. (2015)
	Imaging of brain tumors Jing et al. (2016), Carr et al. (2018)
	Photo-thermal-accoustic therapy for brain tumors Golubewa et al. (2020)
	Anti-tumor gene therapy Staquicini et al. (2020)
	Fluorescence-guided tumor surgery Butte et al. (2014), Cho et al. (2018), Cho et al. (2019)

The versatility of a single nanoscale delivery system to be multiplexed with detection and treatment capabilities defines it as a theranostic (d'Angelo et al., 2019). The ability of a theranostic to visualize tumor cells *in vivo* during surgery and track cellular biodistribution and treatment response throughout the course of adjuvant therapy, can allow for time and cost savings while adopting a personalized medicine approach to improve outcomes (d'Angelo et al., 2019; Mendes et al., 2018). For example, a fluorescent-labelled theranostic NP conjugated to a CD133 monoclonal antibody has recently been shown to enable NIR tracking of patient-derived glioma cancer stem cells in an orthotopic mouse model of glioma (Jing et al., 2016). Finally, a fluorescent-tagged monoclonal antibody targeting the epidermal growth factor receptor (EGFR), cetuximab-IRDye800, has recently been shown in a first-in-human trial demonstrating the ability to identify tumor tissue in glioma patients in real-time under NIR FGS with high correlations to pre-operative gadolinium-enhanced MR imaging and post-operative histopathological staining of tissue specimens (Miller et al., 2018), emphasizing the timeliness and importance of discussing novel brain tumor-targeting theranostic technologies that can potentially surpass the sole reliance of the heterogeneously leaky BBB around high grade tumors allowing for delivery of 5-ALA or ICG.

We previously leveraged the use of M13 bacteriophage (M13 ϕ)—highly specialized and modular nanoscale filamentous protein particles that can be genetically tuned for programmable assembly, chemically modified for surface functionalization of imaging, chemical, or antigenic moieties, for *in vivo* theranostic applications. These applications have included: Tumor-targeted imaging and drug delivery (Ghosh et al., 2012); Image-guided solid tumor resection (Yi et al., 2012; Ghosh et al., 2014); And high contrast short-wavelength infrared (SWIR) fluorescence imaging of vascular and lymphatic structures (Ceppi et al., 2019; Lin et al., 2019). In this mini review and opinion piece, we introduce proof-of-concept data demonstrating our ability to functionalize M13 ϕ for systemic delivery to detect glioma brain tumors using SWIR fluorescence imaging in a patient-derived mouse model of glioma, demonstrating the potential for the use of M13 ϕ as a brain tumor theranostic.

## Results

**FIGURE 1:**
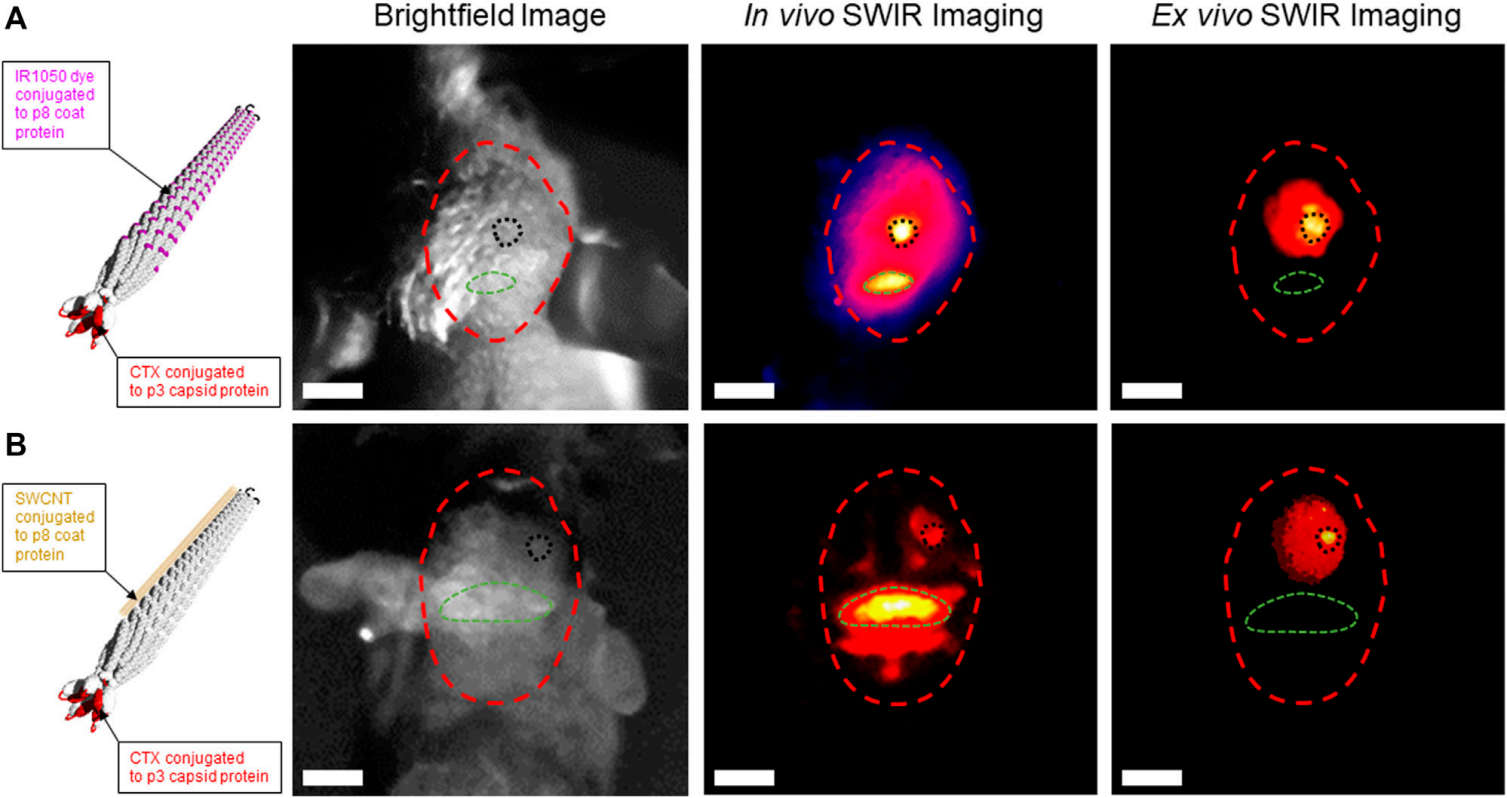
Chlorotoxin (CTX)-functionalized M13 phage conjugated to IR1050 dye or single-wall carbon nanotubes (SWCNT) detect GBM22 human glioma tumors using short-wavelength near infrared II (SWIR) imaging in an intracranial orthotopic xenograft model of human glioblastoma. **(A)** Left panel—schematic of IR1050-CTX-M13 phage. Right panels—brightfield image of the skull of a GBM22 mouse (red dotted circle). *In vivo* SWIR imaging through the skull localizes IR1050-CTX-M13 phage signal at the site of a GBM22 tumor in the right frontal hemisphere (black dotted circle). Background SWIR signal caused by uptake of IR1050-CTX-M13 phage in the occipital bone (green dotted circle). *Ex vivo* SWIR imaging shows uptake of IR1050-CTX-M13 phage in a GBM22 tumor in the mouse brain. **(B)** Left panel—schematic of SWCNT-CTX-M13 phage. *Right panels*—brightfield image of the skull of a GBM22 mouse (red dotted circle). *In vivo* SWIR imaging through the skull localizes SWCNT-CTX-M13 phage signal at the site of a GBM22 tumor (black dotted circle). Background SWIR signal caused by uptake of SWNT-CTX-M13 phage in the occipital bone (green dotted circle). *Ex vivo* SWIR imaging shows uptake of SWCNT-CTX-M13 phage in a GBM22 tumor. White scale bar = 1 cm.

We previously published on our expertise to generate stable rod-like filamentous M13 ϕ with a narrow diameter of 5 nm and length of 900 nm that can be conjugated with various NIR fluorescent dyes or single-wall carbon nanotubes (SWCNT) to enable SWIR imaging of ovarian tumors and lymph nodes in a mouse model of ovarian cancer (Ghosh et al., 2012; Yi et al., 2012; Ghosh et al., 2014; Ceppi et al., 2019). We hypothesized that the extremely narrow diameter and high length-to-diameter aspect ratio of the M13 ϕ would allow it passage across the tight endothelial gap junctions of the BBB if functionalized with a known peptide moiety with proven ability to achieve endocytosis across the BBB with the ability to target glioma tumor cells in the brain following systemic delivery. Several such peptides have been identified including angiopep-2 (Liu et al., 2019), PepC7 (Li et al., 2012), and GL1 (Ho et al., 2010), amongst others, however, there has only been one peptide to-date that has been FDA-approved for in-human use to assist in FGS of higher grade brain tumors—the 36 amino-acid peptide chlorotoxin (CTX), derived from the venom of the *Leiurus quinquestriatus* scorpion (Veiseh et al., 2009; Dardevet et al., 2015; Cohen-Inbar and Zaaroor, 2016). CTX peptide and CTX conjugates have demonstrated the ability to traverse the BBB and achieve binding to over 80% of tumor cells in an intracranial orthotopic model of glioma (Wang et al., 2020). To achieve functionalization of M13 ϕ with CTX, we cloned in the 108 base pair CTX gene sequence upstream of the phage p3 capping protein insertion site. We hypothesized that conjugation of CTX-M13 ϕ with either the NIR II IR1050 dye (Figure 1A, left panel, schematic) or SWCNT (Figure 1B, left panel, schematic) to the p8 coat proteins of M13 ϕ would allow detection of CTX-M13 ϕ at the site of patient-derived GBM22 brain tumors in an intracranial orthotopic xenograft murine model of GBM using SWIR imaging.

Brightfield images of the heads of GBM22 mice show their skulls outlined in red, their occipital boney prominence outlined in green, and the site of intracranial GBM22 tumor induction in the right posterior frontal lobe, outlined in black (Figures 1A,B, right panel, brightfield images). Twenty-four hours following tail vein injection of either IR1050-CTX-M13 or SWCNT-CTX-M13 ϕ, the heads of GBM22 mice were subjected to *in vivo* SWIR imaging (Figures 1A,B, right panel, *In vivo* SWIR images, respectively). Results demonstrated IR1050-CTX-M13 ϕ or SWCNT-CTX-M13 ϕ signal localized to the right posterior frontal lobe region corresponding to the site of GBM22 tumor induction (Figures 1A,B, right panel, *In vivo* SWIR images, black dotted circles). Background uptake of IR1050-CTX-M13 or SWCNT-CTX-M13 phage was observed in the occipital bony protruberance of the mice (Figures 1A,B, right panel, *In vivo* SWIR images, green dotted circles, respectively). To further confirm the uptake of CTX-M13 ϕ at the site of GBM22 brain tumors, we removed the brains from the skulls of GBM22 mice and performed *ex vivo* SWIR imaging. *Ex vivo* SWIR imaging demonstrated focused IR1050-CTX-M13 or SWCNT-CTX-M13 signal at the site of GBM22 tumors (Figures 1A,B, right panel, *Ex vivo* SWIR images, black dotted circles, respectively). The elimination of signal from the bony region corresponding to the occipital bone during *ex vivo* SWIR imaging confirmed the nonspecific uptake of IR1050-CTX-M13 ϕ and SWCNT-CTX-M13 ϕ by the occipital bone (Figures 1A,B, right panel, *Ex vivo* SWIR images, green dotted circles) and the specificity of the CTX-M13 ϕ to detect GBM22 cells in the brain (Figures 1A,B, right panel, *Ex vivo* SWIR images, black dotted circles). Taken together, our preliminary data confirms the modular nature of M13 ϕ to be functionalized and conjugated for use in glioma tumor detection using SWIR imaging with the potential for further development as a theranostic nanomaterial for the treatment of gliomas.

## Discussion

Filamentous bacteriophage are highly stable and scalable nanoscale materials that have been characterized for their inert effects as a nano-carrier for the delivery of a range of vaccine-based therapies in humans (González-Mora et al., 2020; Margot et al., 2020; Chung et al., 2021). Like other filamentous bacteriophage systems, M13 ϕ is genetically tunable and can be manufactured at relative low costs with high uniformity across batches, making this nanoplatform an ideal candidate as a theranostic delivery system. Recent studies have demonstrated the ability of filamentous phage to achieve penetration into the CNS space through intranasal delivery or assisted by temporary disruption of the BBB through convection-enhanced delivery for the treatment of neurological diseases (Carrera et al., 2004; Wan et al., 2009; Dabrowska, 2019).

A recent study using a hybrid vector expressing an adeno-associated virus and a single-stranded M13 phage (AAVP) displaying a double-cyclic RGD4C motif ligand (which targets alpha V integrin receptors expressed on the surface of tumor vascular endothelium) with the gene sequence for the cytotoxic protein tumor necrosis factor (RGD4C-AAVP-*TNF*), demonstrated tumor killing when systemically delivered over 3 consecutive doses in an intracranial orthotopic xenograft mouse model of U87MG human glioma (Staquicini et al., 2020). The same study also leveraged the RGD4C-AAVP construct to express *Herpes simplex* virus thymidine kinase (RGD4C-AAVP-*HSVtk*) in U87MG mice followed by treatment with gangciclovir as suicide gene therapy. Both the RGD4C-AAVP-*TNF* and RGDC4C-AAVP-*HSVtk* constructs achieved the intended cytotoxic and theranostic effects, respectively, demonstrating the potential for using M13 ϕ as a theranostic for the treatment of human gliomas and potentially other brain tumors (Staquicini et al., 2020). Our preliminary data demonstrating the ability of IR1050-CTX-M13 ϕ and SWCNT-CTX-M13 ϕ to detect patient-derived GBM22 tumors in the brains of mice using SWIR imaging (Figures 1A,B, right panel, *Ex vivo* SWIR images), and our previously published studies using NIR II conjugated-M13 ϕ expressing tumor-targeting peptides to detect ovarian cancer tumors during real-time surgical resection in a mouse model of ovarian cancer (Yi et al., 2012; Ghosh et al., 2014), further enforces the potential of using M13 ϕ as a versatile theranostic platform for use in FGS and multimodal treatment of a wide range of tumors, including brain tumors. We are currently conducting further preclinical studies to better characterize the pharmacokinetics, pharmacodynamics, and safety of M13 ϕ in the CNS to assess the translational potential for use in human trials (Tsedev et al., 2022).

The potential for tumor-targeting nanoscale materials to be used in the detection and treatment of a wide range of brain tumors can offer patients a personalized treatment path. In particular, the recent 2021 reclassification of CNS tumors into further molecular subtypes based on genetic modifiers and/or diagnostic molecular pathological features (Louis et al., 2021), has uncovered limitations in using 5-ALA FGS for Gr III glial tumors (Mirza et al., 2022). This study compared outcomes in 69 patients with Gr III gliomas—39 patients had 5-ALA FGS and 30 patients had non-5-ALA FGS. Patients in the 5-ALA group had preoperative MR imaging that demonstrated some degree of contrast enhancement compared to those in the non-5-ALA group. The degree of intraoperative 5-ALA fluorescence directly correlated to the presence of contrast enhancement on preoperative imaging. Interestingly, the degree of intraoperative fluorescence was not associated with either 1p19q codeletion nor IDH mutational tumor status, however, significantly more patients with gliomas that had O-6-methylguanine-DNA methyltransferase methylation received 5-ALA FGS. There were no statistically significant differences in OS nor EoR between groups, but interestingly performance status was worse in the 5-ALA group in the immediate post-operative and 6-month follow-up periods. Patients in the 5-ALA group who had a GTR had significant improvements in OS compared to patients with subtotal resections (STR), however patients who had STRs in the non-5-ALA group had better performance scores at 6 months compared to patients with STRs in the 5-ALA group. This and other studies point towards a potential correlation between molecular subtyping with 5-ALA tumor fluorescence, and argues against the widespread use of 5-ALA FGS in these patients. Similarly, an elegant study by Obaid and colleagues using an NIR-II EGFR-targeting nanoliposome formulation to study the specificity of NP delivery to EGFR-expressing glioma cell lines *in vitro*, *in vivo*, and *ex vivo*, demonstrated that delivery of nanoliposomes to the tumor site did not correlate with EGFR expression in the three different glioma cell lines that were tested, underrepresenting the image-derived molecular specificity by up to 94.2% (Obaid et al., 2021). This suggests that the accumulation of functionalized nanoliposomes (which are the most commonly used types of NPs for clinical applications) at the sites of tumors may be more dependent on the enhanced permeability and retention properties of tumors rather than their specificity of molecular targeting—something that researchers should be aware of when using receptor-ligand biochemistry to improve a nanoparticle’s delivery to the tissue site of interest to assist in FSG. Finally, the ability to tailor theranostics for patients who have subtypes of gliomas that do not respond well to FGS may offer an alternative personalized approach to treating this increasingly heterogeneous group of tumors.

## Conclusion

In conclusion, the increasing use of fluorescence image-guided tumor resection within the neurosurgical community opens an avenue for the introduction of innovative theranostic nanoplatforms that could further assist the tumor surgeon in achieving intraoperative detection and maximal EoR. Whilst future comparative studies are needed to definitively assess the ability of FGS to achieve better patient outcomes compared to the less costly, non-fluorescence-based navigated resection techniques, the potential for theranostics to be applied in a more personalized fashion may lead to breakthroughs in treating complex and heterogeneous brain tumors such as gliomas in order to achieve better survival outcomes afforded by current standard of care treatment regimens, which have largely remained unchanged over the past several decades.
